# Considering Pleistocene North American wolves and coyotes in the eastern *Canis* origin story

**DOI:** 10.1002/ece3.7757

**Published:** 2021-06-05

**Authors:** Paul J. Wilson, Linda Y. Rutledge

**Affiliations:** ^1^ Biology Department Trent University Peterborough ON Canada

**Keywords:** *Canis*, coyotes, eastern wolves, gray wolves, introgressive hybridization, Pleistocene

## Abstract

The evolutionary origins and hybridization patterns of *Canis* species in North America have been hotly debated for the past 30 years. Disentangling ancestry and timing of hybridization in Great Lakes wolves, eastern Canadian wolves, red wolves, and eastern coyotes are most often partitioned into a 2‐species model that assigns all ancestry to gray wolves and/or coyotes, and a 3‐species model that includes a third, North American evolved eastern wolf genome. The proposed models address recent or sometimes late Holocene hybridization events but have largely ignored potential Pleistocene era progenitors and opportunities for hybridization that may have impacted the current mixed genomes in eastern Canada and the United States. Here, we re‐analyze contemporary and ancient mitochondrial DNA genomes with Bayesian phylogenetic analyses to more accurately estimate divergence dates among lineages. We combine that with a review of the literature on Late Pleistocene *Canis* distributions to: (a) identify potential Pleistocene progenitors to southern North American gray wolves and eastern wolves; and (b) illuminate opportunities for ancient hybridization events. Specifically, we propose that Beringian gray wolves (*C. lupus*) and extinct large wolf‐like coyotes (*C. latrans orcutti*) are likely progenitors to Mexican and Plains gray wolves and eastern wolves, respectively, and may represent a potentially unrecognized source of introgressed genomic variation within contemporary *Canis* genomes. These events speak to the potential origins of contemporary genomes and provide a new perspective on *Canis* ancestry, but do not negate current conservation priorities of dwindling wolf populations with unique genomic signatures and key ecologically critical roles.

## Graphical abstract

Eastern wolves may have origins through ancient hybridization between the Beringian wolf and the Pleistocene wolf‐like coyote.

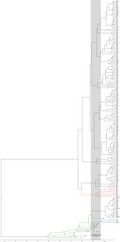

## 1 INTRODUCTION

Almost three decades ago, Wayne and Jenks (1991) proposed a gray wolf–coyote hybrid origin for the endangered red wolf (*Canis rufus*) in the United States. This conclusion, based on early genetic analysis with restriction enzymes and sequencing of the mitochondrial DNA (mtDNA) cytochrome b region, was met with harsh criticism from morphology experts who claimed the hybrid origin hypothesis was inconsistent with the fossil evidence and morphometric analysis of historical skull specimens (e.g., Nowak, 1992). Nine years later, Wilson et al. (2000) used sequencing of the mtDNA control region in combination with microsatellite genotyping of wolves in Algonquin Park, Ontario, to propose the “eastern wolf” (*C*. *lycaon*) as a North American evolved wolf, distinct from gray wolves (*C. lupus*) that originated in Eurasia, but closely related to coyotes (*C. latrans*) and red wolves that are endemic to North America. Since then, analyses of *Canis* evolutionary history have expanded to include genome‐wide single nucleotide polymorphism (SNP) markers and even whole genomes. Researchers claim support for either a two‐species model of *Canis* evolution in North America, whereby all ancestry can be attributed to gray wolves (*C. lupus*) or coyotes (*C. latrans*) (e.g., vonHoldt et al., 2011; vonHoldt, Cahill, et al., 2016) or a three‐species model, wherein ancestry includes a third wolf‐like species unique to eastern North America that encompasses both the eastern and red wolf (*C. lycaon/rufus*) (e.g., Hohenlohe et al., 2017; Rutledge et al., 2015; Rutledge, Wilson, et al., 2012). The overall debate on *Canis* hybridization and evolution is still topical as observed in a recently updated bibliography by vonHoldt & Aardema (2020).

Despite general acceptance of a small eastern wolf with a predisposition for hybridizing with coyotes (Heppenheimer, Brzeski, et al., 2018; Heppenheimer, Harrigan, et al., 2018; Rutledge, Garroway, et al., 2010; Rutledge, White, et al., 2012), there is additional debate on whether the red wolf is part of a larger eastern wolf lineage (assuming support for that model) (Kyle et al., 2008; Rutledge, Wilson, et al., 2012; Wilson et al., 2000), or whether eastern wolves represent coyote‐introgressed red wolves that further hybridized with gray wolves at the northern edge of their historical range (Nowak, 2002). Other *Canis* populations with contentious origins include the following: (a) the Great Lakes wolf (Koblmüller et al., 2009; Leonard & Wayne, 2008) that has been characterized as a gray wolf × coyote “eastern wolf” hybrid (*C. lupus* × *latrans,* vonHoldt, Cahill, et al., 2016; vonHoldt, Kays, et al., 2016), and alternatively as a gray wolf × eastern wolf hybrid (*C. lupus* × *lycaon*, Mech, 2011; Wheeldon & White, 2008); and (b) the eastern coyote that has been described as a Great Lakes gray wolf × coyote hybrid (*C. lupus var*. × *C. latrans*, Kays et al., 2010) and an eastern wolf × coyote hybrid (*C. lycaon* × *latrans*; Rutledge et al., 2015; Rutledge, Garroway, et al., 2010; Wheeldon et al., 2010; Wilson et al., 2009, 2012).

For the most part, these debates focus on the contemporary hybridization between wolves and coyotes and how these interactions do or do not contribute to the origins of eastern North American *Canis*. This paradigm is likely an oversimplification of a complex system of *Canis* evolution. A number of studies have addressed the “enigmatic” nature of eastern wolves and the role of hybridization in their origin, with some explicitly testing or considering a three‐species model (*C. lupus*, *C. latrans*, *C. lycaon*/*rufus*) (Brzeksi et al., 2016; Hailer & Leonard, 2008; Rutledge et al., 2015; Rutledge, Patterson, et al., 2010; and see Heppenheimer et al., 2020; Heppenheimer, Brzeski, et al., 2018; Heppenheimer, Harrigan, et al., 2018; Hohenlohe et al., 2017). Ancestry is, however, frequently tested with the binary lineages of gray wolf and coyote without considering the potentially unique North American ancestry of *C. lycaon*/*rufus* (e.g., Sinding et al., 2018; vonHoldt et al., 2011; vonHoldt, Cahill, et al., 2016; vonHoldt, Kays, et al., 2016). This omission may mask the contribution of this third lineage that is a sister species to coyotes.

Although analysis and genomic simulations of genome‐wide SNPs provided support for the three‐species model (Rutledge et al., 2015), these results could not resolve the possibility that eastern wolves arose from an ancient hybridization event followed by drift (Rutledge et al., 2016; Sefc & Koblmüller, 2016). Typically, little consideration has been given to ancient hybridization models in the origins of eastern *Canis*, with most “ancient” DNA studies focused on early 20th‐century samples (Koblmüller et al., 2009; Wilson et al., 2003) or those from the very late Holocene (350–1900 years ago) (Brzeksi et al., 2016; Rutledge, Bos, et al., 2010). These studies focus on modern forms of wolves and coyotes and not their pre‐Holocene precursors, with some exception in considering the Beringian wolf (Leonard et al., 2007) or where the authors simply recognize the potential for ancient hybridization (Rutledge et al., 2015, 2016; Sefc & Koblmüller, 2016; Sinding et al., 2018). The vast majority of *Canis* hybridization studies fail to consider the fossil‐based morphological studies of Pleistocene and early Holocene *Canis* forms that acknowledge variable morphological characteristics, distributions, and demographic conditions that could facilitate and/or predispose ancient interactions that impact evolutionary processes (Meachen & Samuels, 2012; Meachen et al., 2014, 2016; Nowak, 1979, 2002; Tomiya & Meachen, 2018).

A review of previous and emerging literature reveals a significant range in the models of North American Pleistocene *Canis* evolution. First, there is potential for dispersal of gray wolves (*C. lupus*) from Beringia to more southern distributions inhabited by coyotes (*C. latrans*) and dire wolves (*C. dirus*) prior to the end of the last glacial maxima (LGM: (23–13.5 kya Heintzman et al., 2016)) and North American megafaunal extinctions approximately 13 kya (Heintzman et al., 2016) and 11 kya (Dundas, 1999), respectively. Early pre‐LGM *C. lupus* colonization of southern North America was originally proposed by Vila et al. (1999) with additional supporting genetic (Koblmüller et al., 2016) as well as fossil evidence of Beringian wolves (Leonard et al., 2007) moving south prior to the LGM (Meachen et al., 2016). Recently, Loog et al. (2020) proposed that *C*. *lupus* populations only colonized North America from Beringia starting 15 kya. Second, although there is a paucity of coyote genetic studies considering their Pleistocene history, fossil evidence supports the presence of a wolf‐like coyote (*C. latrans orcutti*) prior to the Holocene, from 40 kya to 11 kya (Meachen & Samuels, 2012; Meachen et al., 2014; Nowak, 1979; Tomiya & Meachen, 2018), although smaller coyotes in southern latitudes, for example, Mexico (Hody & Kays, 2018; Lucas et al., 1997), cannot be excluded. This Pleistocene “coyote” is an important consideration in evaluating the origins of the contemporary Great Lakes, eastern and red wolves as early contact and potential ancient hybridization would have likely consisted of the precursor Beringian *C*. *lupus*, proposed to be an extinct ecotype (Leonard et al., 2007), and the Pleistocene coyote, a wolf‐like coyote that was larger than the modern coyotes that emerged 10 kya (Meachen & Samuels, 2012; Meachen et al., 2014; Tomiya & Meachen, 2018).

In addition to the recent and extensive re‐assessment of *Canis* fossil morphology, the presence of extensive mitochondrial datasets, including ancient *C*. *lupus*, provides an opportunity to more accurately calibrate the timing of species divergence. Accurate dating will allow more robust ancestral inference of critical haplotypes by addressing co‐existence of forms and opportunity for ancient introgression events. Here, we re‐evaluate the origins of contemporary *Canis* species within the framework of mtDNA divergence and from the perspective of Late Pleistocene wolf and coyote distribution. We applied Bayesian approaches to previously published modern and ancient mtDNA datasets to calibrate substitution rates for estimating divergence times (Tong et al., 2018) between wolves and coyotes. We also used phylogenetic analyses to elucidate the presence of ancestral Pleistocene lineages within each species. Overall, we propose a new paradigm to test hypotheses of *Canis* evolution that re‐frames analyses with more accurate divergence times and in consideration of ancient Pleistocene types and their potential interactions.

## 2 MATERIALS AND METHODS

We assessed phylogenetic relationships and divergence times among mitogenome control region haplotypes using Bayesian methods. The software jModelTest 0.1.1 (Posada, 2008) was applied to identify HKY+G as the best substitution model using the Bayesian information criterion for *Canis* control region haplotypes downloaded from GenBank (Figure S1). Sequences were aligned in Geneious using Clustal Omega 1.2.3. Two maximum clade credibility trees were created using BEAST v1.10.4 (Suchard et al., 2018) using time calibrated tips from ancient DNA‐derived haplotypes under a strict clock model, HKY+G substitution model, default optimization schedule, MCMC chain length of 200 million, sampling every 20,000 generations, and removing the first 10% of runs. The two independent runs were combined using the BEAST v1.10.4 package LogCombiner. We analyzed results from BEAST in Tracer v1.7 (Rambaut et al., 2018), and all effective sample sizes (ESS) were much greater than 200, indicating length of MCMC in accurately representing the posterior distribution was appropriate (Kuhner, 2009). The phylogenetic trees we estimated were summarized in the BEAST v1.10.4 package TreeAnnotator and visualized in FigTree 1.4.4 (Rambaut, 2016). Divergence times were calculated as the node heights of the 95% highest posterior density (HPD) intervals.

Modern and ancient whole mitochondrial DNA sequences were downloaded from GenBank (Figure S1) and aligned in Geneious R11.1.4 (Biomatters Ltd.) with ClustalW and default settings (UBC Cost matrix, Gap open cost: 15; Gap extend cost: 6.66). Alignment was trimmed on each end to have the same sequence length and annotated against the domestic dog mtDNA genome (CFU96639). We removed the control region to estimate divergence based on coding regions of the mtDNA genome. We used BEAST 2.6.0 (Bouckaert et al., 2019) to estimate divergence dates and create a phylogenetic tree based on modern and ancient samples. Partitions were assigned as in Loog et al. (2020) with the following three independent mutation models: (a) PCDS1, rRNA, and tRNA with model HKY+I; (b) PCDS2 with model TrN+I; and (c) PCDS3 with model TrN+G. The positions of the partitions were identified based on start codons found from the reference genome annotations. The tree models for the partitions were linked, and the site and clock models were unlinked. Substitution model parameters were set for each partition according to the recommended model. We used a strict clock and added tip dates for the ancient sequences based on the sample ages provided in the source reference. Parameterization of priors was set as described in Loog et al. (2020). Trees were sampled every 5,000 iterations over 50,000,000 iterations, with a burn‐in of 10%. Tracer 1.7.1 was used to assure convergence of parameters, and TreeAnnotator was used to determine the maximum clade credibility consensus tree. The final tree was visualized with FigTree 1.4.4.

We further generated a PHYML tree (Dereeper et al., 2008) using the mitochondrial control region sequences that considered insertion/deletions.

## 3 RESULTS AND DISCUSSION

Pleistocene coyotes and gray wolves have been characterized as morphologically different from their modern forms (Leonard et al., 2007; Meachen & Samuels, 2012; Meachen et al., 2014; Nowak, 1979; Tomiya & Meachen, 2018), with no modern version of dire wolf due to its loss during the megafaunal extinctions (Dundas, 1999). Incorporating Pleistocene forms of gray wolves and coyotes, their associated ancient lineages and their potential interactions, has been limited in framing hypotheses and reconstructing the histories of the eastern wolf, red wolf, and Great Lakes wolf.

Estimates of mitochondrial DNA (mtDNA) divergence have assumed gray wolves and coyotes diverged 1–2 million years ago based on the fossil evidence (Nowak, 1979), an assumption that has carried over into the majority of molecular studies (e.g., Lehman et al., 1991; Rutledge, Patterson, et al., 2010; Vila et al., 1999; Wilson et al., 2000). As a result, a critical first test in reconstructing the population histories of North American *Canis* is calibrating the substitution rates and divergence times of regions of mtDNA with Bayesian‐derived phylogenies that include ancient haplotypes from fossils with reliable carbon dating. A Bayesian phylogeny of whole mitochondrial DNA, minus the control region, that included ancestral sequences and partitioned for different regions and 1st, 2nd, and 3rd positions, derived 940 kya (737, 1,147 95% HPD) for the divergence of gray wolf and coyote (Figure S1). The divergence times of these full mitogenomic sequences are predicted to provide more accurate deeper dating estimates than the single noncoding hypervariable regions used for control region (Duchêne et al., 2011). Our results support the proposed million‐year gray wolf–coyote divergence assumption (e.g., Lehman et al., 1991; Nowak, 1979; Wilson et al., 2000) that has further been validated recently using genome sequencing (Perri et al., 2021).

**FIGURE 1:**
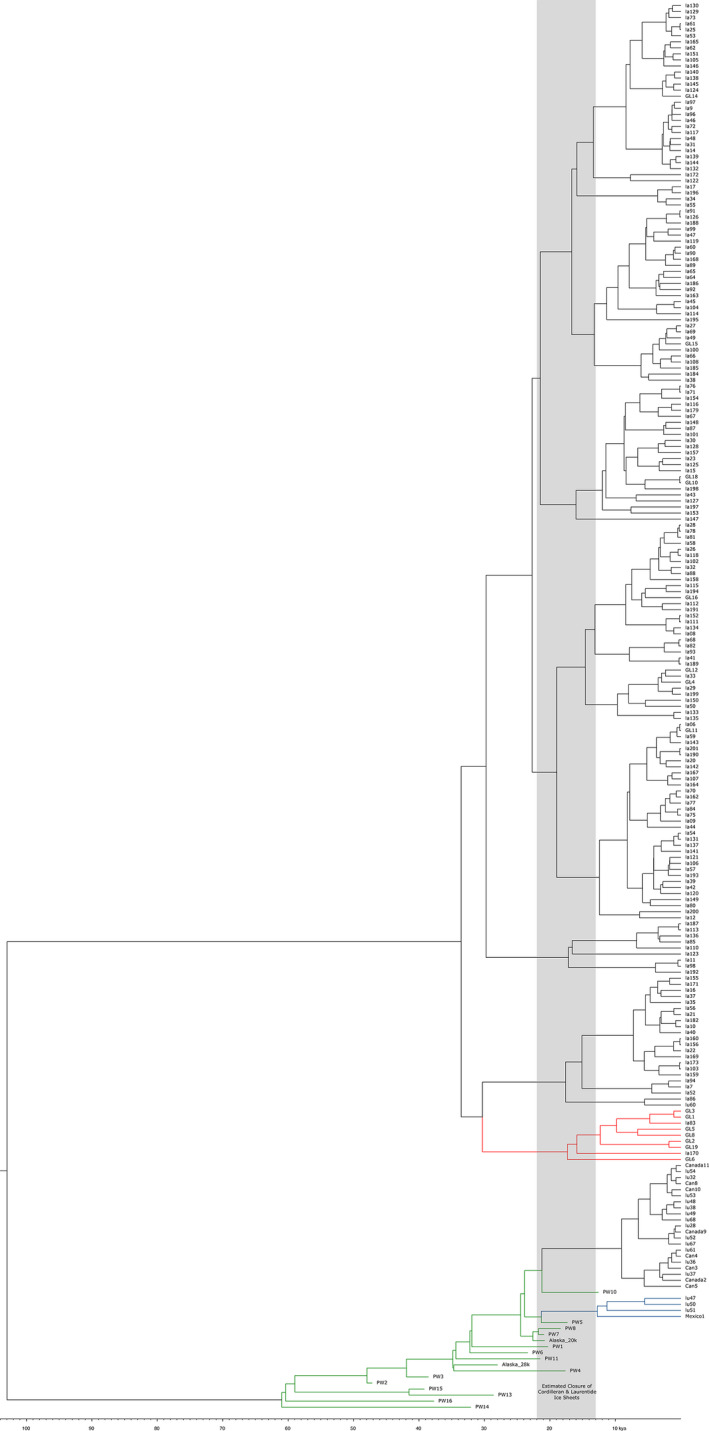
Dated BEAST phylogeny of 405 bp control region. Green branches are ancient Beringian wolf haplotypes, blue branches are Mexican wolf and southern clade haplotypes, and red branches are Great Lakes and eastern wolf haplotypes. The gray area represents the estimated timing the Cordilleran and Laurentide Ice Sheets closed any corridor from Beringia to North America south of the ice sheet 22,000–13,000 years ago

**FIGURE 2:**
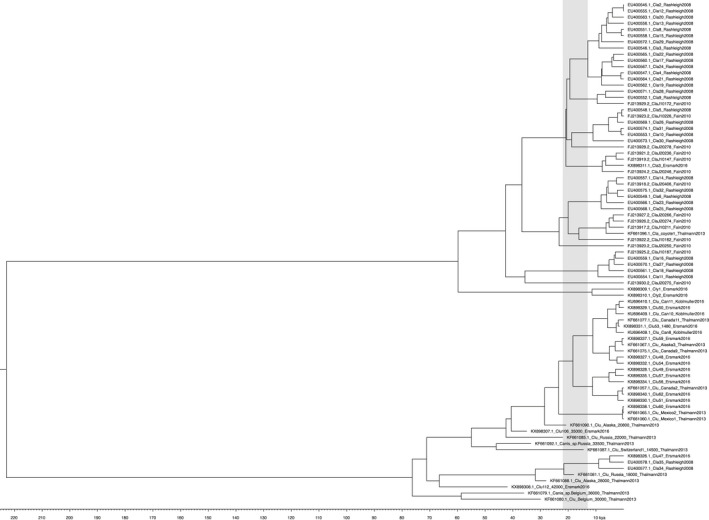
Dated BEAST phylogeny of 550 bp control region. Great Lakes and eastern wolf haplotypes are represented by Cly1 and Cly2 (Ersmark et al., 2016). The gray area represents the estimated timing the Cordilleran and Laurentide Ice Sheets closed any corridor from Beringia to North America south of the ice sheet 22,000–13,000 years ago

**FIGURE 3:**
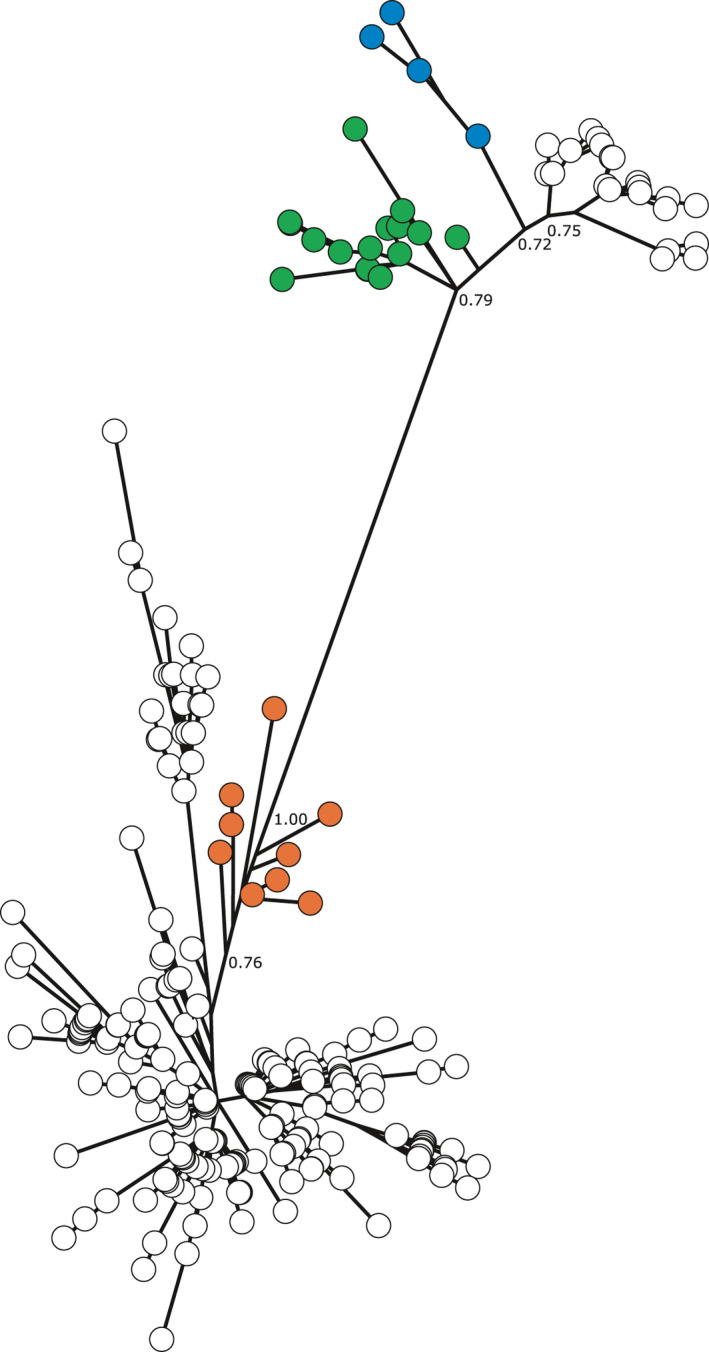
PhyML tree plotted with GrapeTree of 405 bp control region. Green branches are ancient Beringian wolves, blue branches are Mexican wolves, and red branches are Great Lakes and eastern wolves. Posterior probabilities are presented for critical nodes

Due to the absence of eastern and Great Lakes wolf full mitochondrial DNA sequences, for a more complete phylogenetic analysis we focused on two partial control region datasets: a 405 bp dataset (Figure 1) that included ancient samples (Leonard et al., 2007), historical southern US wolf samples (Leonard et al., 2005), and representative eastern wolf/Great Lakes wolf haplotypes (Kays et al., 2010; Leonard & Wayne, 2008), and a 550 bp dataset (Figure 2) (Ersmark et al., 2016; Fain et al., 2010; Rashleigh et al., 2008; Thalmann et al., 2013) that was more limited in representative haplotypes but was assessed for concordance with the shorter control region segment. In general, similar topologies were observed between the two Bayesian analyses, specifically (a) ancestral positioning of Mexican, and southern wolf clades for the 405 bp reconstruction in the *C. lupus* clade; and (b) eastern/Great Lakes haplotypes as ancestral to the remaining *C. latrans* clade. Despite the similar topologies, the posterior probabilities for the 550 bp analysis (Figure S3) were substantially more supportive than the 405 bp analysis (Figure S2). As a result, we applied a PhyML analysis to the 405 bp segment (Figure 3) and confirmed the ancestral positioning of Mexican wolf/southern clade and eastern/Great Lakes wolf haplotypes to *C. lupus* and *C. latrans*, respectively.

As would be predicted, the sequences from ancient specimens (Leonard et al., 2007) were basal to modern gray wolf haplotypes, with one exception: the “southern” clade from early 1900s Mexican wolves (*C. lupus baileyi*) and the Plains wolf (*C. lupus nubilus*) that was flanked by now extinct Beringian wolf haplotypes (Figure 1). The low posterior probability in these relationships limits the interpretation of this result, but the additional analyses presented here at a minimum support the “southern” wolves as ancestral to other modern North American gray wolves. Beringian wolves as an ecotype of gray wolf have purportedly gone extinct (Koblmüller et al., 2016; Leonard et al., 2007), and although their corresponding ancient haplotypes are not seen in contemporary specimens, their role as progenitors to the southern modern wolves (*C. lupus baileyi* and *C. l*. *nubilius*) cannot be excluded. A similar basal position of the Mexican wolf was observed with the 550 bp sequence and has been consistently observed to be the most ancestral North American gray wolf (Sinding et al., 2018; Thalmann et al., 2013; Vila et al., 1999); its lineage originated approximately 25–30 kya (Figure 2) consistent with Koblmüller et al. (2016) prior to the closure of the Ice Free Corridor between the Cordilleran and Laurentide ice sheets during the LGM.

**FIGURE 4:**
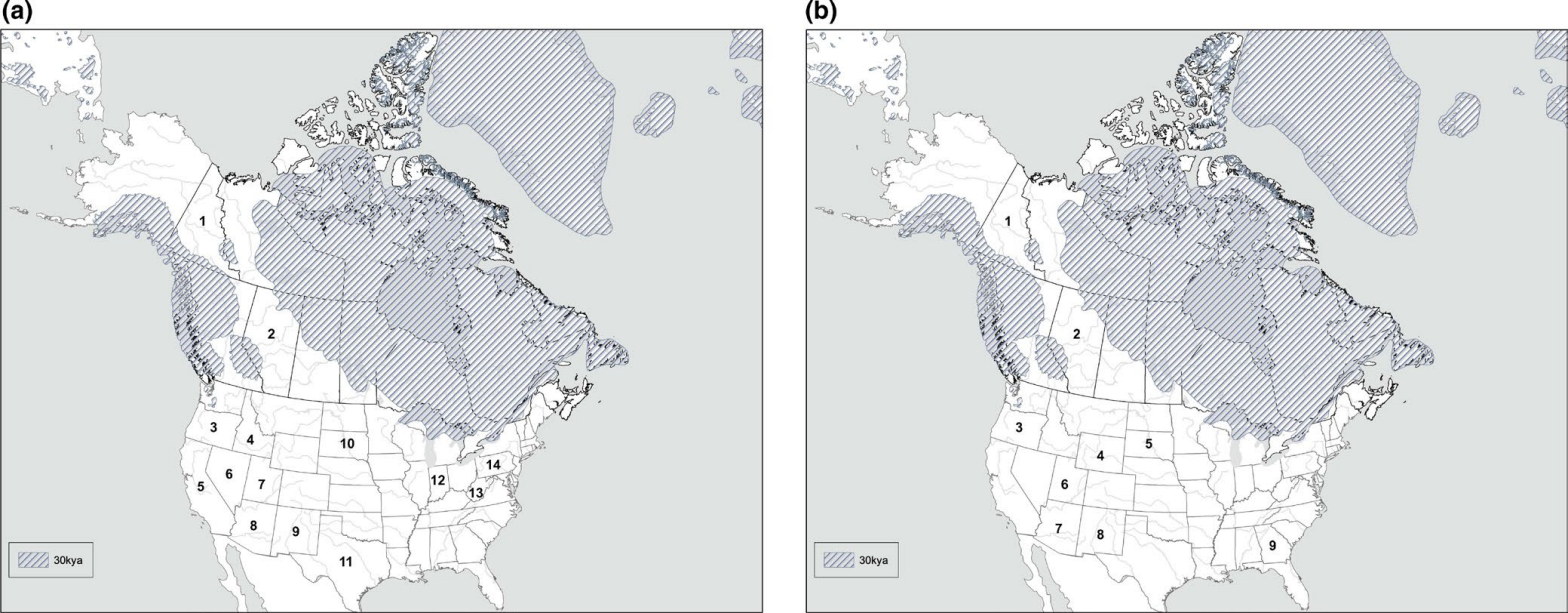
Distribution of *Canis latrans* (a) and *lupus* (b) Faunmap fossils through the Rancholabrean with maximum ice sheets estimated at 30,000 years ago (ya) (Batchelor et al., 2019). (a) Distribution of *Canis latrans* fossils by province/state and minimum and maximum age estimates: (1) YU: 47,170–47,170 ya; (2) AB: 36,800–39,000 ya; (3) OR: 35,000–65,000 ya; (4) ID: 21,000–33,000 ya, 21,000–33,000 ya, 75,000–125,000 ya, 15,000–72,000 ya, 58,000–86,000 ya; (5) CA: 23,000–27,000 ya, 27,000–34,000 ya, 26,000–32,000 ya, 30,000–35,000 ya, 67,000–112,000 ya, 40,000–110,000 ya; (6) NV: 40,000–110,000 ya; (7) UT: 40,000–100,000 ya; (8) AZ: 31,000–110,000 ya; (9) NM: 13,000–25,000 ya, 13,500–20,000 ya, 25,000–35,000 ya, 25,000–35,000 ya, 20,120–25,000 ya; (10) SD: 26,075–26,075 ya; (11) TX: 25,000–35,000 ya, 31,400–35,000 ya, 23,230–23,230 ya; (12) IN: 24,390–25,710 ya; (13) WV: 17,060–29,400; and (14) PA: 13,740–13,740 ya; 11,000–11,000 ya. (b) Distribution of *Canis lupus* fossils by province/state and minimum and maximum age estimates: (1) YU: 20,780–49,400 ya, 30,500–34,000 ya; 27,270–28,570 ya; (2) AB: 25,960–44,800 ya; (3) OR: 35,000–65,000 ya; (4) WY: 12,777–15,500 ya, 15,500–20,250 ya, 13,500–27,000; (5) SD: 26,075–26,075 ya; (6) UT: 14,500–18,000 ya; (7) AZ: 31,000–110,000 ya; (8) NM: 13,000–25,000 ya, 13,500–20,000 ya, 15,030–30,000 ya, 25,000–35,000 ya, 25,000–35,000 ya, 20,120–25,000 ya; and (9) GA: 13,560–24,080 ya

The role of the Beringian wolf in modern southern gray wolf (e.g., Mexican and Plains wolf; Leonard et al., 2005) evolution in North America is further supported by the recent fossil evidence from the Natural Pit site in Wyoming (Meachen et al., 2016). Evidence suggests these wolves colonized the south through the ice‐free corridor dividing the Cordilleran and Laurentide ice sheets before the last glacial maximum (LGM) and the maximal closure prior to 21 kya (Kleman et al., 2010) to 23 kya (Heintzman et al., 2016). Multiple waves of gray wolf colonization in southern regions have been proposed, particularly in the evolution of the Mexican wolf (Thalmann et al., 2013; Vila et al., 1999). However, a single pre‐LGM colonization event was interpreted from analysis of whole mtDNA genomes, with the proposal that modern gray wolves evolved south of the LGM and colonized north following the reformation of the ice‐free corridor to Beringia (Koblmüller et al., 2016). Recognition of the distinctiveness of the Mexican wolf was provided, with speculation that these wolves represent a mixture of other evolving gray wolves south of the maximal ice sheets. In contrast, and based on a broader dataset, Loog et al. (2020) proposed that modern gray wolves colonized North America from Beringia starting 15 kya. However, the basal ancestral position of Mexican wolves to other North American gray wolves, dating to the pre‐LGM period of 30–35 kya in our analyses, was left as an open question in Loog et al. (2020) with the recognition of potential earlier colonization. Regardless of the role Beringia had as a cradle for modern *C. lupus* evolution, there is strong evidence of ancestral wolves south of the LGM that are likely candidates as being the progenitor of the southern wolf clade (Leonard et al. 2005). This association of the historical Mexican (*C. lupus baileyi*) and Plains wolf (*C. lupus nubilus*) clade with ancient Beringian wolf haplotypes (Leonard et al., 2007; Figure 2) further supports the southern pre‐LGM movement of Pleistocene gray wolves through an open glacial corridor earlier than 23 kya. Interestingly, the distribution of FAUNMAP Rancholabrean (240–11 kya) *Canis* fossils (Figure 4a,b) of gray wolf specimens (Figure 4b) prior to and into the LGM (Figure 1) largely mapped to New Mexico and Wyoming, where the proposed corridor to the Natural Pit site is located (Meachen et al., 2016). This distribution pattern is concordant with the proposed distribution of the Mexican and Plains wolves (Leonard et al., 2005; Nowak, 2002).

Whereas fossil and genetic evidence support a southern presence of the Pleistocene “Beringian” gray wolf, it is postulated that its distribution was constrained by the presence of the dire wolf (Meachen & Samuels, 2012; Meachen et al., 2016; Tomiya & Meachen, 2018) until the megafaunal extinctions approximately 11 kya (Dundas, 1999). The Pleistocene coyote (*C. latrans orcutti*), a larger, more wolf‐like canid than contemporary coyotes was regionally sympatric with dire wolves: with contemporary coyotes representing the most recent 10,000 years of the species evolutionary history. The coalescence of coyote‐like mtDNA was approximately 30–60 kya in our Bayesian phylogenetic analyses of the control region, which suggests that the most ancestral lineages would correspond to the *C. latrans orcutti* subspecies that pre‐date contemporary coyote lineages. Interestingly, the most basal *C. latrans* clade dating to the pre‐LGM period was those sequences found in eastern wolves and Great Lakes wolves (Figures 1, 2 Cly1 and 2). This well‐defined clade supports an ancestral lineage to the eastern and Great Lakes wolves through *C. l. orcutti*, but it does not reject the introgression of modern coyote haplotypes within the range of natural variation of the species as hybridization with modern coyotes and the *C. latrans* wolves (i.e., eastern wolves) has most likely occurred in both contemporary and historic times (Wilson et al., 2000, 2003, 2009).

The implication of the basal eastern wolf clade during the timeframe *C. latrans orcutti* was on the western North American landscape is that this more wolf‐like animal is the progenitor to the modern eastern wolves. During the Pleistocene, *C. l. orcutti* unlikely inhabited eastern geographies and was more limited to western North America, and it was not until the terminal Pleistocene that the east was re‐occupied by a small wolf (Nowak, 2002). This re‐occupation of eastern North America by a small wolf‐like canid coincides with a shift from the more wolf‐like *C. l. orcutti* into the modern version of the coyote (Meachen & Samuels, 2012). The proposal that *C. l. orcutti* was the progenitor to the eastern wolves is not unprecedented in that Young and Goldman (1944) considered larger Pleistocene coyotes as far west as California to be red wolves.

Two extant Pleistocene lineages south of the LGM, one *C. lupus* and one *C. latrans*, raise the question whether these species had opportunity for ancient hybridization that may have maintained or even facilitated wolf‐like characteristics in a transition from *C. latrans orcutti* to *C. lycaon/rufus*. Recent genomic characterization has estimated the proportion of gray wolf and coyote admixture in the North American canids as gray wolf:coyote proportions of 70:30 for the Great Lakes wolf and eastern wolf combined, 30:70 for the red wolf, and 90:10 for the Mexican wolf (Sinding et al., 2018); similar proportions for one or more of these combinations have also been estimated elsewhere (vonHoldt et al., 2011; vonHoldt, Cahill, et al., 2016; vonHoldt, Kays, et al., 2016). This evidence supports introgressive hybridization but typically this is interpreted in the context of modern inter‐breeding (with some exception, see Sefc & Koblmüller, 2016; Sinding et al., 2018). These studies applied the SABER analytical software to genome‐wide SNP and whole genomes (vonHoldt et al., 2011; vonHoldt, Cahill, et al., 2016). This approach, however, is limited in its ability to detect multiple hybridization events (e.g., past vs. recent) (Supple & Shapiro, 2018). Furthermore, the gradient of gray wolf‐to‐coyote ancestry may be expanded in that the pooling of eastern wolves, typically from Algonquin Provincial Park, whereas Great Lakes wolves may not be appropriate (see Hohenlohe et al., 2017; Rutledge, Wilson, et al., 2012). Previous work shows Algonquin Park wolves to have significantly less gray wolf genetic signal than wolves from northern Ontario and the Great Lakes states (Rutledge, Garroway, et al., 2010; Wilson et al., 2009). Regardless, these findings support the opportunity for ancient hybridization between the Beringian wolf and the Pleistocene wolf‐like coyote.

This hypothesis of Pleistocene hybridization is further supported by genetic evidence. A signature of potential ancestral mtDNA introgression may be associated with haplotype lu60, a coyote haplotype found in the Mexican wolf (Leonard et al., 2005). This lineage diverged in the Pleistocene (18 kya; Figure 1), a time that pre‐dates modern coyotes, when *C. latrans orcutti* inhabited the western landscape. The lu60 haplotype is related to a single observed coyote sequence (la86) found only in Texas, a geographic region that overlaps part of the historical range of the Mexican wolf (Hendricks et al., 2016). The absence of lu60 and highly similar sequences in modern coyotes further supports a more ancient event, particularly given the maintenance of a high contemporary haplotypic diversity in extant coyotes. Furthermore, surveys of interspecific gene flow among *Canis* identified support for ancient hybridization, including the following: (a) introgression from the Mexican wolf lineage into coyotes (Gopalakrishnan et al., 2018) and vice versa (Sinding et al., 2018); (b) the generation of novel population‐specific alleles in eastern wolves (Sinding et al., 2018; vonHoldt, Cahill, et al., 2016) including differentiation between Great Lakes and eastern wolves (Sinding et al., 2018); and (c) relatively consistent levels of wolf versus coyote genetic makeup in Great Lakes and eastern wolves (Sinding et al., 2018) supporting a more historical introgression event. Although this evidence does not reject the more recent hybridization that has clearly taken place (e.g., Wilson et al., 2000, 2003), these contemporary signatures also support our proposed ancient hybridization between Pleistocene coyotes and Beringian wolves that could have contributed to modern introgressive signatures.

Resolving the hypothesis of ancient hybridization between Beringian wolves and Pleistocene coyotes and the impact on the ancestry of contemporary North American *Canis* requires genetic and morphometric data from additional ancient specimens (e.g., Beringian wolf skulls and *C. latrans orcutti*, respectively, from the Wyoming Natural Trap site, Meachen et al., 2016). Although fossil evidence supports the pre‐LGM southward movement of Beringian wolves, there is also some evidence to suggest opportunities for northward movement of *C. latrans orcutti*. More specifically, a 47 kya fossil from the Yukon has been identified morphologically as a “coyote” (Figure 4). A Beringian distribution of coyotes is further evident in the coyote ancestry within Siberian canids, with higher proportions in 48‐ to 50‐kya‐year‐old specimens than more recent, that is, 14–17 kya, skulls (Ramos‐Madrigal et al., 2021). Expansion on the existing ancient DNA dataset associated with Beringian wolves (Leonard et al., 2007) by obtaining more specimens and/or expanding into genomic‐based markers would further refine the evolutionary story and relationship of Pleistocene *Canis* species. Overall, the distribution of Pleistocene wolves and coyotes south of the Cordilleran/Laurentide ice sheets during the LGM, and signatures of older introgression, support the likelihood that ancient hybridization has shaped the ancestry of extant wolves and coyotes in eastern regions of North America, where contemporary hybridization patterns have muddied ancestry patterns based on nuclear genome scans.

These inferences provide a new perspective that could reshape our understanding of North American *Canis* ancestral origins. This current perspective is based purely on the timing of divergence and not on the ecological adaptation and/or speciation of modern eastern wolves and coyotes. Given the different niches occupied by eastern wolves and coyotes, along with the potential association of modern eastern wolves with the more wolf‐like *C. latrans orcutti*, we suggest the common name “wolf” is most appropriate for eastern wolves, regardless of admixture with ancient Beringian wolves or modern gray wolves. The taxonomic nomenclature, given differential hybridization with gray wolves and coyotes, depending on the eastern wolf in question, is significantly more complex, and more targeted research will be required to move beyond both the binary modern gray wolf × coyote hybridization commonly utilized (Sinding et al., 2018; vonHoldt et al., 2011; vonHoldt, Cahill, et al., 2016) and distinct species lineage (Rutledge et al., 2015; Wilson et al., 2000). Furthermore, future research should include contributions from *C. dirus* into the contemporary *Canis* genome complex, a task which is now accessible with the publication of the *C. dirus* genome (Perri et al., 2021). This would also apply to the role smaller southern North American Pleistocene coyotes (Hody & Kays, 2018; Lucas et al., 1997) had in the evolution of modern coyotes.

By combining a review of recent fossil evidence and Pleistocene *Canis* distributions with a re‐analysis of existing ancient and modern mitochondrial DNA data, we have introduced a more inclusive evolutionary framework based on potential ancient interactions by which to test hypotheses of North American *Canis* ancestry. Based on our assessment, future research should consider several specific aspects to improve our understanding of *Canis* species origins, some being re‐iterated from the literature while others debated in the literature:

1. Gray wolves appear to have colonized the southern distribution of the United States prior to the LGM before the ice sheets closed from 23 to 13 kya;
2. The Beringian wolf may well have been the ecotype that was the progenitor to the southern wolf clade of the Mexican wolf (*C. lupus baileyi*) and potentially the Plains wolf (*C. lupus nubilus*);
3. The “coyote” on the landscape during the later Pleistocene was a larger more wolf‐like animal *C. latrans orcutti*, with evidence presented here that it is the potential progenitor to the North American line of eastern wolves;
4. Ancient hybridization, that is, prior to the Holocene (11 kya), may have involved the Beringian wolf and the large wolf‐like Pleistocene coyote. Interbreeding seems possible given the wolf‐like nature of both forms compared with today's more divergent morphological forms where natural viable gray wolf × coyote hybridization in western regions is largely absent; and
5. North American *Canis* operate along a range of hybrid ancestries contributed to both contemporary and ancient inter‐breeding.

Overall, more fully considering Pleistocene progenitors (specifically the Beringian wolf as progenitor to southern US wolves; and *C. l. orcutti* as the progenitor of eastern wolves) south of the LGM raises the complexity of modern *Canis* taxonomy beyond a strictly 2‐ or 3‐species model. For example, from a strictly species‐level perspective, Pleistocene gray wolves and coyotes south of the LGM would represent an ancient 2‐species model with a high probability of introgressive hybridization, with a modern 3‐species model that saw the emergence of the modern coyote and contemporary hybridization. Although this current study does not resolve the question of the number and nomenclature of eastern North American wolf types, it is nonetheless an important step to refocus a decades‐long unresolved debate on the evolution of North American wolves.

## CONFLICT OF INTEREST

The authors declare that they have no conflict of interest.

## AUTHOR CONTRIBUTIONS

**Paul J. Wilson:** Conceptualization (equal); Formal analysis (equal); Methodology (equal); Software (equal); Visualization (equal); Writing‐original draft (equal); Writing‐review & editing (equal). **Linda Y. Rutledge:** Conceptualization (equal); Formal analysis (equal); Investigation (equal); Methodology (equal); Software (equal); Validation (equal); Visualization (equal); Writing‐original draft (equal); Writing‐review & editing (equal).

## Supporting information

Fig S1

Fig S2

Fig S3

Appendix S1

## Data Availability

All sequence data are available through the GenBank public data repository. GenBank accessions for DNA sequences used in the control region and whole mtDNA genome analysis can be found in the Appendix S1.
